# Associations between Visual, Hearing Functioning and Cognitive Functioning among Hispanics/Latinos

**DOI:** 10.1093/geroni/igab046.3566

**Published:** 2021-12-17

**Authors:** David Lee, D Diane Zheng, Laura McClure, Karen Cruickshanks, Charlotte Joslin, Hector Gonzalez, Neil Schneiderman, Byron L Lam

**Affiliations:** 1 University of Miami Miller School of Medicine, Miami, Florida, United States; 2 University of Miami Miller School of Medicine, University of Miami Miller School of Medicine, Florida, United States; 3 McPherson Eye Research Institute, University of Wisconsin-Madison, Madison, Wisconsin, United States; 4 University of Illinois at Chicago, Chicago, Illinois, United States; 5 University of California, San Diego, La Jolla, California, United States; 6 University of Miami, Miami, Florida, United States

## Abstract

Findings that visual impairment (VI) and hearing impairment (HI) are associated with cognitive functioning are drawn from studies that involved few Hispanic/Latino participants. We utilized data from the Miami Ocular SOL ancillary study to the Hispanic Community Health Study/Study of Latinos (HCHS/SOL) with 1056 participants aged 45 and older. The outcomes were neurocognitive performances assessed by the Digit Symbol Substitution Test (DSST, executive function), Word Frequency Test (verbal fluency), Brief Spanish-English Verbal Learning Test-recall (B-SEVLT recall, episodic memory), and the Six-Item Screener (global cognitive functioning). Visual functioning was measured by National Eye Institute Visual Function Questionnaire (NEI-VFQ). Hearing function which was measured by Hearing Handicap Inventory for Adults and Elderly (HHIA/HHIE) was available for all HCHS/SOL participants (n=9343). Multiple regression was performed for each cognitive outcome while controlling for age, gender, education, Hispanic/Latino ethnicity background, cardiovascular risk factors, depression and complex design. NEI-VFQ was associated with 3 of the 4 cognitive outcomes: DSST (β =0.14, se=0.027, p<0.01), Word Frequency Test (β=0.042, se=0.016, p<0.01), B-SEVLT-recall (β=0.021, se=0.007, p<0.03). HHIA/HHIE was not associated with any of the cognitive measures examined. The HHIA/HHIE analysis was repeated using data from all sites; similar results were observed. Visual functioning but not hearing functioning is associated with worse cognition in Hispanics/Latinos, although previous HCHS/SOL analysis indicated that hearing loss as assessed by pure tone audiometry was associated with worse cognitive functioning. Longitudinal assessment of both clinical and functional measures is needed to understand the impact of sensory impairment on cognition in Hispanics/Latinos.

